# Lipid Peroxidation, Nitric Oxide Metabolites, and Their Ratio in a Group of Subjects with Metabolic Syndrome

**DOI:** 10.1155/2014/824756

**Published:** 2014-06-02

**Authors:** Gregorio Caimi, Rosalia Lo Presti, Maria Montana, Davide Noto, Baldassare Canino, Maurizio R. Averna, Eugenia Hopps

**Affiliations:** Dipartimento Biomedico di Medicina Interna e Specialistica, Università di Palermo, Via del Vespro 129, 90100 Palermo, Italy

## Abstract

Our aim was to evaluate lipid peroxidation, expressed as thiobarbituric acid-reactive substances (TBARS), nitric oxide metabolites (nitrite + nitrate) expressed as NO_*x*_, and TBARS/NO_*x*_ ratio in a group of subjects with metabolic syndrome (MS). In this regard we enrolled 106 subjects with MS defined according to the IDF criteria, subsequently subdivided into diabetic (DMS) and nondiabetic (NDMS) and also into subjects with a low triglycerides/HDL-cholesterol (TG/HDL-C) index or with a high TG/HDL-C index. In the entire group and in the four subgroups of MS subjects we found an increase in TBARS and NO_*x*_ levels and a decrease in TBARS/NO_*x*_ ratio in comparison with normal controls. Regarding all these parameters no statistical difference between DMS and NDMS was evident, but a significant increase in NO_*x*_ was present in subjects with a high TG/HDL-C index in comparison with those with a low index. In MS subjects we also found a negative correlation between TBARS/NO_*x*_ ratio and TG/HDL-C index. Considering the hyperactivity of the inducible NO synthase in MS, these data confirm the altered redox and inflammatory status that characterizes the MS and suggest a link between lipid peroxidation, inflammation, and insulin resistance, evaluated as TG/HDL-C index.

## 1. Introduction

The metabolic syndrome (MS) is characterized by an elevated cardiovascular morbidity and mortality [1].

A strong association between MS and oxidative stress has been demonstrated [2, 3]. The end products of lipid peroxidation, which refers to the oxidative degradation of lipids resulting in cell membrane damage, are malondialdehyde (MDA), 4-hydroxynonenal (HNE), and 4-oxy-2-nonenal (ONE), obtained by the oxidation of polyunsaturated fatty acids. Other systemic markers of lipid peroxidation are oxidized low-density lipoprotein (oxLDL), isoprostanes, and thiobarbituric acid-reactive substances (TBARS), which are increased in MS subjects [4–10]. Plasma lipid peroxidation products levels are higher in subjects with 5 MS components compared with those with 2 or 3 MS components [11]. A multivariate analysis, performed by Leiva et al., showed that elevated levels of TBARS are associated with a 21-fold risk for the development of MS [12]. OxLDLs are increased in newly diagnosed MS subjects [13] and are also considered a predictive biomarker for MS [14]. OxLDLs are significantly correlated with coronary artery calcium measured using computerized tomography and with greater internal carotid intima-media thickness in MS subjects [15]. Recently, in diabetic subjects with coronary artery disease, elevated serum MDA-modified LDL (MDA-LDL) levels have been observed and, in subjects with more than 4 MS criteria, a higher MDA-LDL/LDL-cholesterol ratio has been found. MDA-LDLs are correlated with lipid profile, C-reactive protein (CRP), and adiponectin levels [16]. Also the urinary 8-epi-prostaglandin F2*α* (8-epi-PGF2*α*) is increased and significantly correlated with CRP in MS subjects, underlining the close link between oxidative stress and systemic inflammation [17].

Recently in a group of MS subjects we observed a marked increase of the nitric oxide metabolites (NO_*x*_), not associated with the presence of diabetes mellitus (DM) [18]. Our data were in agreement with other papers [19–21] that described a NO_*x*_ increase in MS. The explanation of this finding is ascribed to the degree of inflammation accompanying MS that induces the expression of inducible nitric oxide synthase (iNOS) by macrophages. As it is known, in addition, the iNOS is activated by cytokines, such as TNF-*α*, IL-1*β*, and interferon [22–24], even if in literature there are contrasting data regarding the direct relationship between NO_*x*_ and some of these cytokines [25–28].

In this reexamination of our group of MS subjects, we calculated also the TBARS/NO_*x*_ ratio that, up to now, has been evaluated in preeclampsia [29], in juvenile essential hypertension [30], and in hypertensive adolescents with obesity or uraemia [31, 32]. In MS the TBARS/NO_*x*_ ratio may be considered as an integrated marker of plasma lipid peroxidation and degree of inflammation.

In this study, we evaluated lipid peroxidation, expressed as TBARS, nitric oxide metabolites (NO_*x*_), and their ratio (TBARS/NO_*x*_) in a group of MS subjects subdivided according to the presence or not of diabetes mellitus and also according to the degree of insulin resistance, expressed as triglycerides/HDL-cholesterol (TG/HDL-C) index; this index, in fact, may be considered an indicator of insulin resistance [33–35] its logarithm being inversely correlated with insulin sensitivity measures, such as the homeostasis model assessment and the quantitative insulin sensitivity check index [36].

## 2. Subjects and Methods

We enrolled 106 subjects (45 women and 61 men, mean age: 53.5 ± 8.9 years) with MS defined according to the International Diabetes Federation criteria [37]. Subsequently, MS subjects were subdivided, respectively, into diabetics (DMS) (14 women and 29 men) and nondiabetics (NDMS) (31 women and 32 men) and into MS subjects with low (27 women and 26 men) or with high (18 women and 35 men) TG/HDL-C ratio. These two last subgroups of 53 subjects were selected according to the median value of the TG/HDL-C ratio. The control group consisted of 41 subjects (14 women and 27 men, mean age: 41.6 ± 7.9 years) selected from the hospital staff; control subjects were free of medical diseases as assessed by clinical history, physical examination, electrocardiography, and routine hematological and urine analysis. In all the participants cholesterol and triglycerides were measured by standard enzymatic procedures, high-density lipoprotein (HDL) cholesterol after phosphotungstic acid/magnesium chloride precipitation and enzymatic determination of cholesterol, and low-density lipoprotein (LDL) cholesterol was determined by the Friedewald formula. Mean and S.D. of age, anthropometric profile, glycometabolic pattern, lipid profile, and blood pressure values of the entire group and of the four subgroups of MS subjects (subdivided according to the presence of DM or to the TG/HDL-C ratio) are shown in Tables 1 and 2.

At fasting blood samples were collected by venous puncture from the antecubital vein of each subject and immediately transferred to glass tubes anticoagulated with EDTA-K3; then we evaluated lipid peroxidation and NO metabolites.

### 2.1. Lipid Peroxidation

The oxidation of polyunsaturated fatty acids was determined in plasma by the detection of the thiobarbituric acid-reactive substances (TBARS) generated by peroxidative processes, which include lipid peroxides and MDA. The evaluation of TBARS was made by fluorimetry, using the 1,1,3,3-tetramethoxypropane as standard [38].

### 2.2. NO Metabolites

Considering that in vivo NO has a very short life (less than 0.1 sec) and it is converted into nitrite (NO_2_^−^), which has a half-life of few minutes, and into the more stable nitrate (NO_3_^−^), NO_*x*_ represents almost only the nitrate concentration. In the laboratory method adopted by us at first nitrate was converted into nitrite by a nitrate reductase, and then nitrite was assessed by spectrophotometry after the addition of the Griess reagent [39].

### 2.3. Statistical Analysis

The values were expressed as means ± S.D. The difference between the control group and MS subjects was evaluated according to Student's *t*-test for unpaired data. The comparison between control group and MS subjects subdivided according to the presence or not of DM and according to the TG/HDL-C index was performed using the one-way analysis of variance (ANOVA), integrated with Bonferroni's multiple posttest. The values of TBARS and NO_*x*_ and of TBARS/NO_*x*_ ratio were correlated with the age, the anthropometric profile, the blood pressure values, and the glycometabolic and lipid pattern using the linear regression test. The null hypothesis was rejected for *P* values less than 0.5.

## 3. Results

Examining the age, the anthropometric profile, the blood pressure values, and the glycometabolic pattern of MS subjects subdivided according to the presence or not of DM, we observed (Table 2) that age, waist circumference, and blood glucose levels were higher in DMS, while total cholesterol and LDL-cholesterol were higher in NDMS.

Examining the same parameters in MS subjects subdivided according to the TG/HDL-C index (Table 2), we observed that total cholesterol and triglycerides were increased in the subgroup with high TG/HDL-C index and that the HDL-cholesterol was increased in the subgroup with low TG/HDL-C index.

In the entire group of MS subjects an increase in TBARS and in NO_*x*_ levels and a marked decrease in TBARS/NO_*x*_ ratio were evident in comparison with normal controls (Table 3). In the control group, as well as in the whole group of MS subjects, no statistical correlation between TBARS and NO_*x*_ was observed (data not shown). Subdividing MS subjects into DMS and NDMS (Table 4) and also according to the low or high TG/HDL-C index (Table 5), we found an increase in TBARS and in NO_*x*_ levels and a decrease in TBARS/NO_*x*_ ratio in all the subgroups compared with normal controls. Employing the Bonferroni posttest, instead, we found no significant difference between DMS and NDMS regarding TBARS, NO_*x*_, and TBARS/NO_*x*_ ratio (Table 4), while we noted a significant increase in NO_*x*_ in subjects with a high TG/HDL-C index in comparison with those with a low index (Table 5).

**Figure 1:**
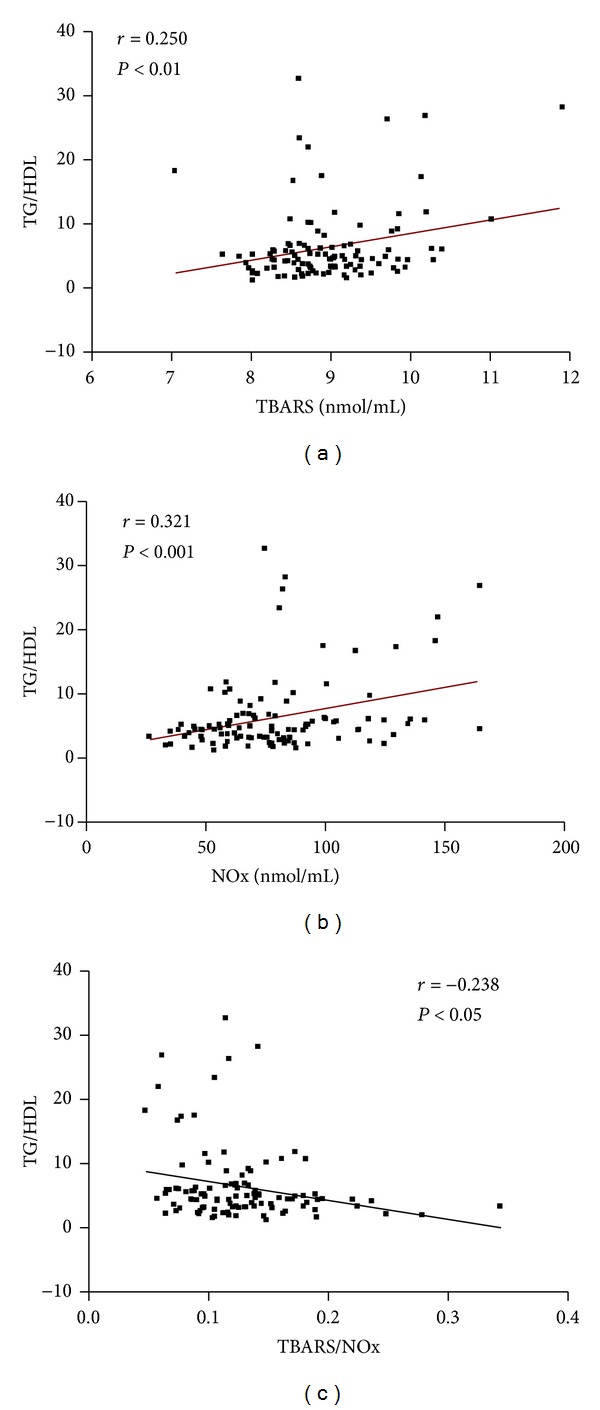
Correlations between TG/HDL index and, respectively, TBARS, NO_*x*_, and TBARS/NO_*x*_ ratio in MS patients.

In the entire group of MS subjects we found a positive correlation between TBARS and TG/HDL-C index (*r* = 0.250; *P* < 0.01) and between NO_*x*_ and TG/HDL-C index (*r* = 0.321; *P* < 0.001) and a negative correlation between TBARS/NO_*x*_ ratio and TG/HDL-C index (*r* = −0.238; *P* < 0.05) (Figure 1).

Examining the linear regression among TBARS, age, anthropometric profile, blood pressure values, and glycometabolic pattern, in the entire group of MS subjects we observed only a positive correlation between TBARS and fasting blood glucose level (*r* = 0.206; *P* < 0.02).

Examining the linear regression among NO_*x*_ and all the parameters previously considered, in the entire group of MS subjects we found only a positive correlation between NO_*x*_ and triglycerides (*r* = 0.344; *P* < 0.001).

Examining instead the linear regression among TBARS/NO_*x*_ ratio and the above-mentioned characteristics of the MS subjects, we found in the whole group only a negative correlation between TBARS/NO_*x*_ and triglycerides (*r* = −0.259; *P* < 0.007).

## 4. Discussion

The analysis of the data concerning the evaluation of lipid peroxidation, nitric oxide metabolites, and their ratio in MS shows some aspects that deserve to be underlined.

The first point concerns the asymmetrical increase in TBARS and NO_*x*_ in the entire group of MS subjects; in fact, the TBARS increase is of 52.2% while the NO_*x*_ increase is of 184.4%; therefore, the TBARS/NO_*x*_ ratio is markedly decreased (−64.7%) in comparison with normal controls. This asymmetrical behavior might explain the lack of correlation between TBARS and NO_*x*_ observed by us, differently from others, who found a positive correlation between hydroperoxide levels and NO_*x*_ in MS [40]. In this regard, it must be considered that we evaluated a parameter of lipid peroxidation (TBARS) that includes not only hydroperoxides but also malondialdehyde.

The second point concerns the different behavior of TBARS, NO_*x*_, and TBARS/NO_*x*_ ratio in MS subjects subdivided according to the presence or not of DM or according to the TG/HDL-C index. In fact, the subdivision of MS subjects into DMS and NDMS does not show any difference in these parameters, while the subdivision according to the TG/HDL-C index shows a difference in NO_*x*_ between low TG/HDL-C index subjects and high TG/HDL-C index subjects. This behavior is imputable to the marked increase in NO_*x*_ observed in the subgroup where a condition of greater insulin resistance is evident.

The third point that needs to be investigated is the bidirectional role played by the insulin resistance in lipid peroxidation and NO_*x*_. The pivotal role of the insulin resistance in MS is well known and the results observed in this research seem to confirm this assumption. In the entire group of MS subjects a positive correlation between TBARS and TG/HDL-C index and between NO_*x*_ and TG/HDL-C index is evident, suggesting a close link between the degree of insulin resistance, the lipid peroxidation, and the synthesis of NO_*x*_. In other clinical conditions that are included among the principal criteria for MS, such as obesity [25] and arterial hypertension [41], a significant correlation between NO_*x*_ and insulin levels was found. However, no correlation between NO_*x*_ and insulin resistance (evaluated as steady state plasma glucose) has been observed in DM subjects [42]. In nondiabetic hypercholesterolemic subjects a positive correlation between the urinary 8-epi-PGF2*α* and the HOMA-IR was observed [43]. From our data we observed an interesting negative correlation between TG/HDL-C index and TBARS/NO_*x*_ ratio, which underlines how the reduction of TBARS/NO_*x*_ ratio is associated with the degree of insulin resistance.

All the aspects described in this third point call the attention to the link between lipid peroxidation, iNOS activity, and insulin resistance. In animal models [44, 45] the iNOS seems to play an indirect role in the pathogenesis of the insulin resistance in peripheral tissues, and in particular in the skeletal muscle. Similarly, recent research on experimental models has underlined how some bioproducts of lipid peroxidation, such as HNE and ONE, are able to induce structural and functional changes in human insulin [46] and how HNE causes insulin resistance [47]. The role played by lipid peroxidation in the insulin resistance development has been suggested also in sedentary adults [48], in which lipid peroxides seem to act on the skeletal muscle. Recently [49] other authors have hypothesized that HNE and oxysterols may be the link between the adipose tissue dysfunction and the abnormality of glucose homeostasis.

In agreement with our data, some authors observed in DM subjects a correlation between TBARS and fasting glucose level [50]. Differently from others, who found a positive correlation between oxLDL, age, systolic blood pressure, and body mass index (BMI) [51], a positive correlation between MDA and BMI [52], and a positive correlation between lipoperoxides and systolic blood pressure [53], we did not note any correlation between TBARS, age, anthropometric parameters, and blood pressure values. In this research no correlation between TBARS, total cholesterol, LDL-cholesterol, and HDL-cholesterol was noted, while we observed a negative correlation between TBARS and triglycerides, considered by us occasional and physiologically inexplicable. Other authors in hypercholesterolemic subjects found a positive correlation between MDA, total cholesterol, and LDL-cholesterol and a negative correlation between MDA and HDL-C [54]. Regarding the NO_*x*_ we observed neither correlation between NO_*x*_ and age nor correlation between NO_*x*_, anthropometric profile, and blood pressure values, differently from other authors [55–57]. In addition, we did not find any correlation between NO_*x*_ and lipid profile, although some significant correlations between NO_*x*_ and lipid parameters have been observed in adolescent subjects [58]. The positive correlation between NO_*x*_ and triglycerides found by us in these subjects agrees with that reported by other authors in postmenopausal women with MS [21] and in healthy population [56], but it has not been observed in normal-weight obese syndrome [28].

From the analysis of the data reported in this study, the data reported in this study clearly confirmed the abnormality of the oxidative status accompanying the MS, as previously described by us in the same group of MS subjects, in which we have examined the behavior of NO_*x*_ [18], and also the protein oxidation [59] and the total antioxidant status (in press).

## 5. Conclusions

In conclusion, the simultaneous examination of TBARS, NO_*x*_, and TBARS/NO_*x*_ ratio in MS subjects, subdivided, respectively, according to the presence of DM or to the TG/HDL-C index, shows a different trend of these parameters in relation to the subdivision criteria and, in particular, a significant positive association between NO_*x*_ and the degree of insulin resistance. This datum, like other aspects discussed in the text, might suggest considering the possible use of the antioxidant treatment in MS subjects in order to attenuate the insulin resistance that seems to affect the peripheral tissues, and in particular the skeletal muscle. In this regard, up to now, several molecules, such as vitamins C and E and flavonoids [49], *α*-lipoic acid, *α*-tocopherol, glutathione, N-acetylcysteine, coenzyme Q10, and taurine [60], have been employed to prevent or to treat MS and associated diseases.

## Figures and Tables

**Table 1:** 

	Mean ± S.D.	Range
Waist circumference (cm)	106.7 ± 11.2	86–135
Body mass index (Kg/m^2^)	32.21 ± 4.53	22.5–45.2
Systolic blood pressure (mmHg)	132.1 ± 16.3	100–210
Diastolic blood pressure (mmHg)	81.2 ± 9.9	50–110
Fasting glucose (mg/dL)	114.3 ± 44.3	68–347
Total cholesterol (mg/dL)	213.9 ± 53.0	106–390
HDL-cholesterol (mg/dL)	40.4 ± 10.8	14–80
LDL-cholesterol (mg/dL)	133.2 ± 46.5	52.4–292.0
Triglycerides (mg/dL)	220.2 ± 147.8	52–759
TG/HDL index	6.399 ± 6.033	1.060–32.52

Means ± S.D. and range of the anthropometric profile, blood pressure values, and metabolic pattern in all MS subjects.

MS: metabolic syndrome.

**Table 2:** 

	MS patients withoutDM	MS patients withDM	MS patients with low TG/HDL index	MS patients with high TG/HDL index
Waist circumference (cm)	102.8 ± 8.8	114.4 ± 11.7^§^	106.7 ± 11.16	106.8 ± 11.46
Body mass index (Kg/m^2^)	31.5 ± 4.2	33.2 ± 5.0*	32.45 ± 4.55	31.94 ± 4.55
Systolic blood pressure (mmHg)	130.0 ± 13.3	136.0 ± 20.5	133.2 ± 18.8	131.1 ± 13.7
Diastolic blood pressure (mmHg)	82.2 ± 8.9	79.6 ± 11.4	80.5 ± 10.9	81.94 ± 8.9
Fasting glucose (mg/dL)	92.2 ± 10.3	147.5 ± 54.2^§^	114.5 ± 39.7	114.2 ± 48.7
Total cholesterol (mg/dL)	228.0 ± 48.3	193.1 ± 53.1^§^	201.2 ± 40.8	226.6 ± 60.6^†^
HDL-cholesterol (mg/dL)	39.7 ± 9.3	41.4 ± 12.8	46.9 ± 9.3	33.9 ± 8.1^‡^
LDL-cholesterol (mg/dL)	147.9 ± 45.8	112.8 ± 39.7^§^	123.5 ± 37.5	143.0 ± 58.7
Triglycerides (mg/dL)	231.0 ± 145.9	204.2 ± 150.9	137.4 ± 42.6	302.9 ± 168.4^‡^
TG/HDL index	6.739 ± 6.190	5.900 ± 5.832	3.00 ± 0.95	9.80 ± 7.00^‡^

Means ± S.D. of the anthropometric profile, blood pressure values, and metabolic pattern in MS patients subdivided, respectively, into nondiabetics and diabetics and into patients with low and high TG/HDL index.

*P < 0.05; ^§^ *P* < 0.001 versus MS patients without DM (Student's *t*-test).

^†^ *P* < 0.05; ^‡^ *P* < 0.001 versus MS patients with low TG/HDL index (Student's *t*-test).

MS: metabolic syndrome.

DM: diabetes mellitus.

**Table 3:** 

	Control subjects	All MS patients
TBARS (nmol/mL)	5.902 ± 1.211	8.983 ± 0.722^§^
NO_*x*_ (nmol/mL)	28.07 ± 18.83	79.82 ± 29.22^§^
TBARS/NO_*x*_ ratio	0.363 ± 0.311	0.128 ± 0.049^§^

Means ± S.D. of TBARS, NO_*x*_, and TBARS/NO_*x*_ ratio in control subjects and in all MS patients.

^§^ *P* < 0.001 versus control subjects (Student's *t*-test).

MS: metabolic syndrome.

TBARS: thiobarbituric acid-reactive substances.

NO_*x*_: nitric oxide metabolites (nitrite + nitrate).

**Table 4:** 

	Control subjects	MS patients without DM	MS patients with DM	*F*
TBARS (nmol/mL)	5.902 ± 1.211	8.834 ± 0.628^§^	9.200 ± 0.801^§^	185.8^1^
NOx (nmol/mL)	28.07 ± 18.83	80.99 ± 33.93^§^	78.10 ± 20.76^§^	55.1^1^
TBARS/NO_*x*_ ratio	0.363 ± 0.311	0.129 ± 0.057^§^	0.125 ± 0.033^§^	28.5^1^

Means ± S.D. of TBARS, NO_*x*_, and TBARS/NO_*x*_ ratio in control subjects and in MS patients subdivided into nondiabetics and diabetics.

^1^ *P* < 0.001 (ANOVA).

^§^ *P* < 0.001 versus control subjects (Bonferroni's test).

MS: metabolic syndrome.

DM: diabetes mellitus.

TBARS: thiobarbituric acid-reactive substances.

NO_*x*_: nitric oxide metabolites (nitrite + nitrate).

**Table 5:** 

	Control subjects	MS patients with low TG/HDL index	MS patients with high TG/HDL index	*F*
TBARS (nmol/mL)	5.902 ± 1.211	8.902 ± 0.581^§^	9.063 ± 0.838^§^	179.6^1^
NO_*x*_ (nmol/mL)	28.07 ± 18.83	72.67 ± 27.34^§^	86.97 ± 29.54^§#^	61.9^1^
TBARS/NO_*x*_ ratio	0.363 ± 0.311	0.140 ± 0.057^§^	0.115 ± 0.035^§^	28.9^1^

Means ± S.D. of TBARS, NO_*x*_, and TBARS/NO_*x*_ ratio in control subjects and in MS patients subdivided according to the TG/HDL index.

^1^ *P* < 0.001 (ANOVA).

^§^ *P* < 0.001 versus control subjects (Bonferroni's test).

^#^ *P* < 0.05 versus MS patients with low TG/HDL index (Bonferroni's test).

MS: metabolic syndrome.

TBARS: thiobarbituric acid-reactive substances.

NO_*x*_: nitric oxide metabolites (nitrite + nitrate).
